# Short-term outcomes of traction-assisted versus conventional endoscopic submucosal dissection for superficial gastrointestinal neoplasms: a systematic review and meta-analysis of randomized controlled studies

**DOI:** 10.1186/s12957-019-1639-z

**Published:** 2019-06-04

**Authors:** Mengting Xia, Yunfeng Zhou, Jiajie Yu, Wenwen Chen, Xiaotao Huang, Juan Liao

**Affiliations:** 1 Department of Gastroenterology, Affiliated Hospital of North Sichuan Medical College, Nanchong, Sichuan China; 2 Departments of Thoracic Surgery, West China School of Public Health and West China Fourth Hospital, Sichuan University, Chengdu, Sichuan People’s Republic of China; 3 Chinese Evidence-Based Medicine Center, West China Hospital, Sichuan University, Chengdu, Sichuan China; 4 Departments of Gastroenterology, West China School of Public Health and West China Fourth Hospital, Sichuan University, 16#, Section 3, Renmin Nan Lu, Chengdu, 610041 Sichuan People’s Republic of China

**Keywords:** Endoscopic submucosal dissection (ESD), Traction, Superficial gastrointestinal neoplasms, Meta-analysis

## Abstract

**Background:**

In recent years, some traction-assisted approaches have been introduced to facilitate endoscopic submucosal dissection (ESD) procedures by reducing the procedure time and risks related to the procedure. However, the relative advantages of traction-assisted endoscopic submucosal dissection (T-ESD) are still being debated. This study aimed to assess the efficacy of T-ESD for the treatment of superficial gastrointestinal neoplasms.

**Methods:**

We searched MEDLINE, Embase, and Cochrane library up to March 31, 2019 for randomized controlled trials (RCTs) comparing T-ESD and conventional endoscopic submucosal dissection (C-ESD) for superficial gastrointestinal neoplasms. The main endpoints are en bloc resection, complete resection, procedure time, perforation, and delayed bleeding. Pooled risk ratio (RR), Peto odds ratio (OR), and mean difference (MD) were calculated to compare T-ESD and C-ESD. This study is registered with PROSPERO, number CRD42018108135.

**Results:**

A total of 7 RCTs with 1007 patients were included in this meta-analysis. There were no significant differences between the T-ESD and C-ESD groups in the pooled estimate of en bloc resection, complete resection, and delayed bleeding (RR = 1.00, 95% CI 0.99, 1.01, *I*^2^ = 0%, *P* = 0.66; RR = 1.00, 95% CI 0.98, 1.03, *I*^2^ = 0%, *P* = 0.81; OR = 0.95, 95% CI 0.48, 1.86, *I*^2^ = 19%, *P* = 0.87,respectively). The pooled estimate indicated that the procedure time was significantly shorter in the T-ESD group (MD = − 16.19, 95% CI − 29.24, − 3.13, *I*^2^ = 87%, *P* = 0.02) than in the C-ESD group. Compared to C-ESD, T-ESD was associated with lower incidence of perforation (OR = 0.32, 95% CI 0.11, 0.91, *I*^2^ = 0%, *P* = 0.03).

**Conclusions:**

T-ESD is a safe and effective treatment option with a low perforation rate and shorter procedure time than C-ESD for superficial gastrointestinal neoplasms. Future multi-center (including European populations), randomized controlled trials of larger sample size and long-term outcomes of T-ESD are required.

## Introduction

Superficial gastrointestinal (GI) neoplasms are defined as lesions limited to the mucosa or submucosa without invading the muscularis propria, regardless of the presence of lymph node involvement. GI neoplasms include esophageal neoplasms, gastric neoplasms, duodenum neoplasms, and colorectal neoplasms. With the development and widespread implementation of endoscopic techniques, such as chromoendoscopy, magnifying endoscopy, magnifying narrow-band imaging, and confocal microscopy, the diagnosis rates of patients with superficial GI neoplasms have been increasing [1–4]. Early diagnosis and therapy of GI neoplasms will greatly improve the quality of life and survival rates. There are several treatment options for GI neoplasms, such as the endoscopic mucosal resection (EMR) [5], endoscopic submucosal dissection (ESD) [6], and surgical resection of the tumor and regional lymph nodes through laparoscopic or open operation [7].

The endoscopic treatment of EMR was initially introduced for gastric neoplasms, and subsequently, for esophageal neoplasms and colorectal neoplasms. It has been widely accepted as the standard treatment for superficial gastrointestinal neoplasms because of its minimal invasiveness. However, it is difficult to complete the en bloc resection, and this difficulty results in low curative resection and high local recurrence. To overcome this problem, ESD was developed for superficial gastrointestinal neoplasms, and it has been rapidly adopted all over the world.

However, the universal adoption of ESD has been limited by its long procedure time and high risk of complications, such as perforation and bleeding [8–10]. To improve the ESD procedure by facilitating visualization of the submucosal layer and maintaining good maneuverability, traction-assisted endoscopic submucosal dissection (T-ESD) was proposed. In 2005, Saito et al. described the traction device of the sinker system for the first time to promote the ESD procedure [11]. Recently, various strategies of traction have been developed, such as clip with line [12], external grasping forceps [13], and internal traction [14], but the efficacy of these strategies remain obscure. We, therefore, conducted this meta-analysis of randomized trials to assess the efficacy of T-ESD vs conventional ESD (C-ESD) for the treatment of superficial gastrointestinal neoplasms.

## Methods

We followed the reporting standards set by Preferred Reporting Items for Systematic reviews and Meta-Analyses (PRISMA) [15].

### Eligibility criteria

We included RCTs that included a comparison of T-ESD vs C-ESD for patients with superficial gastrointestinal neoplasms and that explicitly reported data on at least one of the outcomes: en bloc resection, complete resection, procedure time, perforation, or delayed bleeding. We excluded duplicate publications, non-English studies, and studies lacking clinical endpoints data.

### Literature search

We searched MEDLINE, Embase, and Cochrane Library from inception to March 31, 2019. The search strategy combined MeSH terms and free-text regarding “endoscopic submucosal dissection” and “traction.” Full-search strategies are provided in the Appendix. The reference list of included articles was checked to identify additional relevant studies.

### Study process

A pair of reviewers (MTX and YHZ) independently screened titles/abstracts for potential eligibility and full texts for final eligibility; assessed the risk of bias; and collected data from each eligible trial using standardized, pilot tested forms. The reviewers resolved disagreements through discussion or adjudication by a third reviewer (WWC).

### Risk of bias of assessment

We assessed the risk of bias of RCTs using the Cochrane tool [16], including random sequence generation, allocation concealment, blinding of participants and personnel, blinding of outcome assessment, incomplete outcome data, selective reporting, and other bias.

### Data extraction

We collected the following information from each eligible RCT: study characteristics (first author, year of publication, country, and number of patients); patient characteristics (age, tumor size, and location), intervention (method of traction), and outcome data (en bloc resection, complete resection, procedure time, perforation, and delayed bleeding). Data that were reported as median (range) were converted to mean ± SD according to the methodology of Hozo et al. [17].

### Statistical analysis

RevMan 5.3 was used to analyze the data extracted from every study. We analyzed RCTs using risk ratio (RR) for dichotomous outcomes and mean difference (MD) for continuous outcomes. For the outcomes with low event rate (< 5%), we pooled data using Peto’s method. We reported the pooled effects and their associated 95% confidence intervals (CIs). A *P* value less than 0.05 was considered statistically significant. We examined statistical heterogeneity among studies using the *I*^2^ statistic as well as Cochrane’s chi-square test. If *I*^2^ > 50%, that indicated significant heterogeneity, and a random effects model was used.

For each meta-analysis, we explored sources of heterogeneity with the subgroup hypotheses: type of patient (gastric cancer vs colorectal cancer vs esophageal cancer). We tested the subgroup difference using an interaction test.

## Results

### Study characteristics

**Fig. 1:**
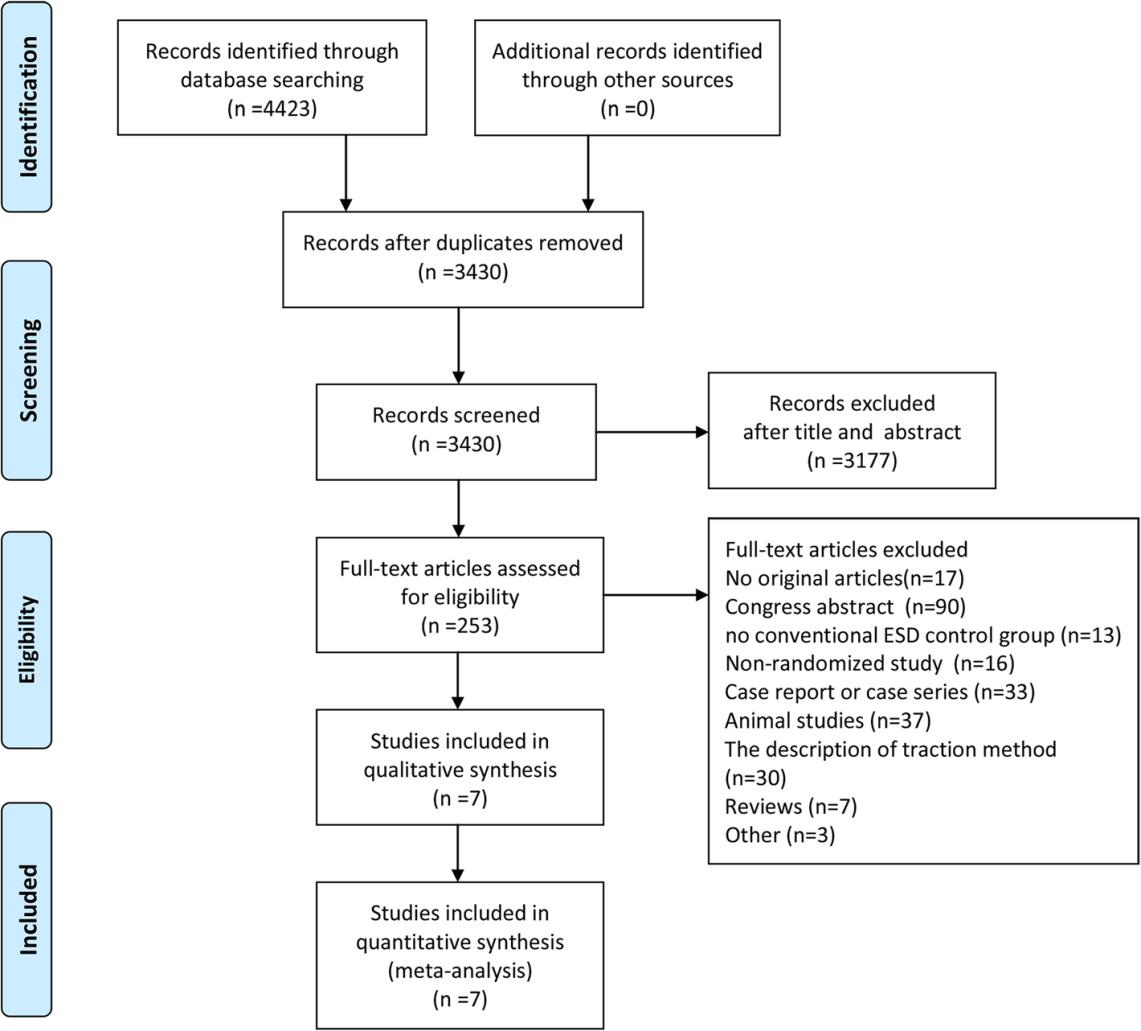
Flow diagram of study selection

We identified 4423 articles by searching databases (Fig. 1). Of these, 993 were excluded as duplicates, and 3177 articles were excluded based on the exclusion criteria. After two reviewers independently read the full text, 7 studies [18–24] with a total of 1007 patients were eventually included. The sample size of the study populations described in the included articles varied from 40 to 635. For three of the studies, the lesions were located in the colorectum, for three studies, they were located in the stomach, and for one study, it was located in the esophagus. Six of these studies were performed in Japan, and one in Korea. The study characteristics are summarized in Table 1.

**Table 1:** characteristics of included studies

Author, year of publication	Country	Patients (*n*) (T-ESD vs C-ESD)	Age (years) (T-ESD vs C-ESD)	Location of lesions	Inclusion criteria for lesions size (mm) (T-ESD vs C-ESD)	Methods of traction
Ahn et al. [18] 2013	Korea	26:25	66.5 ± 8.8 vs 62.9 ± 8.7	Gastric neoplasm	20.5 ± 7.9 vs 19.4 ± 6.5	Transnasal endoscope
Ritsuno et al. [21] 2014	Japan	27:23	66.2 ± 9.6 vs 66.4 ± 8.9	Colorectal tumors	33.5 ± 12.5 vs 37.8 ± 13.1	S–O clip
Koike et al. [19] 2015	Japan	20:20	71 ± 6.3 vs 69.5 ± 9.5	Esophageal carcinoma	24(11–92) vs 27(8–48)	Clip with thread
Mori et al. [20] 2017	Japan	21:22	74 ± 10 vs 72 ± 12	Colorectal tumors	NA	Ring-shaped thread
Yamasaki et al. [22] 2018	Japan	42:42	65(41–84) vs 67(43–86)	Colorectal neoplasm	30(20–55) vs 30(20–60)	Clip-and-thread
Yoshida et al. [23] 2018	Japan	319:316	70.2 ± 9.4 vs 71 ± 8.4	Gastric neoplasms	15.7 ± 10.1 vs 15.5 ± 8.9	Dental floss clip
Ban et al. [24] 2018	Japan	49:55	71.2 ± 6.5 vs 69.0 ± 9.5	Gastric cancers or gastric adenomas	NA	Clip-flap

*T-ESD* traction-assisted endoscopic submucosal dissection; *C-ESD* conventional endoscopic submucosal dissection; *NA* not available

Age and size of lesions was expressed with (mean ± SD) or median (range)

### Risk of bias assessment

The risk of bias in the included studies was rigorously assessed. Of the seven RCTs, five trials [18, 19, 22–24] described the specific methods used for random sequence generation, one study [20] used the parity method, and one study [21] just mentioned “random.” Three studies [19–21] concealed the treatment allocation; one study [23] did not conceal the allocation to the patients and operators, and three studies [18, 22, 24] did not mention concealment. One study [23] was not blinded to the patients and operators, and one study [22] was not blinded to the operators. In one study [18], nine patients dropped out. All of the studies avoided selective outcome reporting. Details of the methodological approach are shown in Table 2.

**Table 2:** The risk of bias of the included studies

Author	Random sequence generation	Allocation concealment	Blinding of participants and personnel	Blinding of outcome assessment	Incomplete outcome data	Selective reporting	Other bias
Ahn et al. [18] 2013	Low risk	Unclear	Unclear	Unclear	High risk	Low risk	Low risk
Ritsuno et al. [21] 2014	Unclear	Low risk	Unclear	Unclear	Low risk	Low risk	Low risk
Koike et al. [19] 2015	Low risk	Low risk	Unclear	Unclear	Low risk	Low risk	High risk
Mori et al. [20] 2017	Unclear	Low risk	Unclear	Unclear	Low risk	Low risk	Unclear
Yamasaki et al. [22] 2018	Low risk	Unclear	High risk	Unclear	Low risk	Low risk	Unclear
Yoshida et al. [23] 2018	Low risk	High risk	High risk	Unclear	Low risk	Low risk	Unclear
Ban et al. [24] 2018	Low risk	Unclear	Unclear	Unclear	Low risk	Low risk	High risk

### Quantitative synthesis

#### En bloc resection

**Fig. 2:**
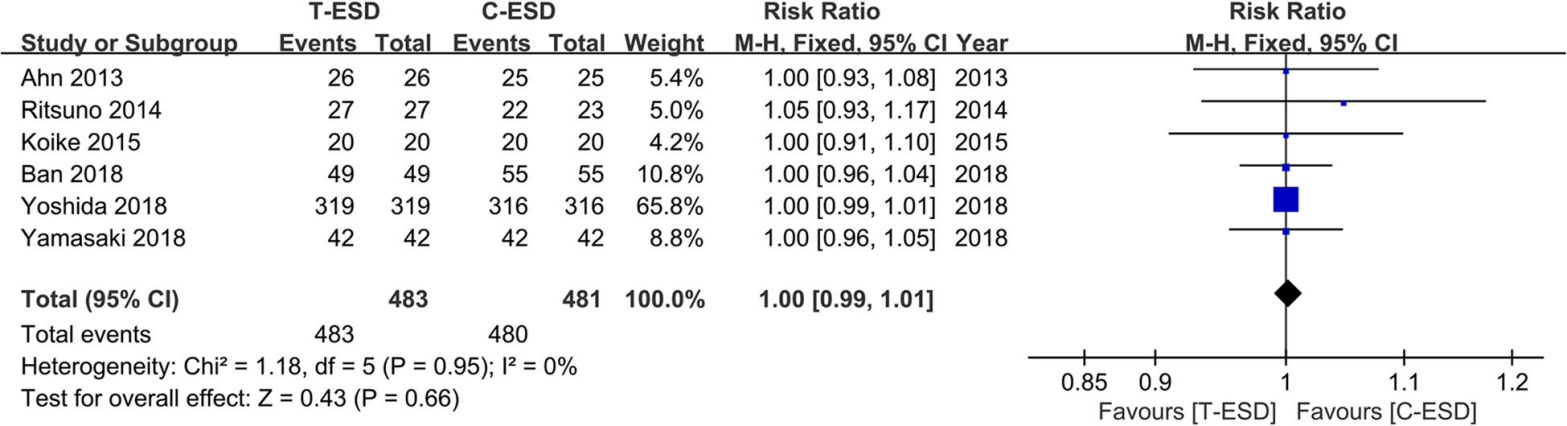
Forest plot of en bloc resection

Six studies, including 964 patients, reported data on en bloc resection. The pooled estimate of en bloc resection on the fixed effects model indicated no significant difference in the T-ESD and C-ESD groups (RR = 1.00, 95% CI 0.99, 1.01, *I*^2^ = 0%, *P* = 0.66). There was no statistical heterogeneity (Fig. 2).

#### Complete resection

**Fig. 3:**
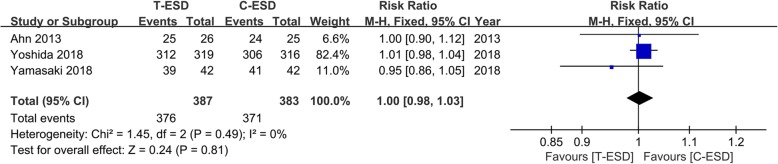
Forest plot of complete resection

Three studies, including 770 patients, reported complete resection. The pooled estimate of complete resection on the fixed effects model indicated no significant difference in the T-ESD and C-ESD groups (RR = 1.00, 95% CI 0.98, 1.03, *I*^2^ = 0%, *P* = 0.81). There was no statistical heterogeneity (Fig. 3).

#### Procedure time

**Fig. 4:**
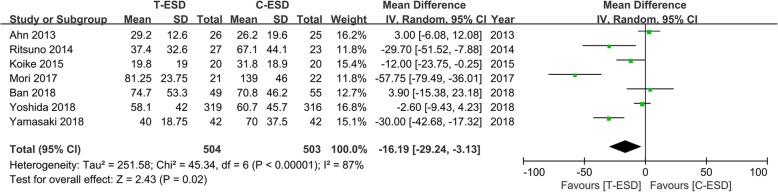
Forest plot of procedure time

The procedure time was reported in all studies, including 1007 patients. The pooled estimate on the random effects model indicated that the procedure time was significantly shorter in the T-ESD group than in the C-ESD group (MD = − 16.19, 95% CI − 29.24, − 3.13, *I*^2^ = 87%, *P* = 0.02). The heterogeneity was significant (Fig. 4).

#### Perforation

Perforation data were reported in all studies, including 1007 patients.

**Fig. 5:**
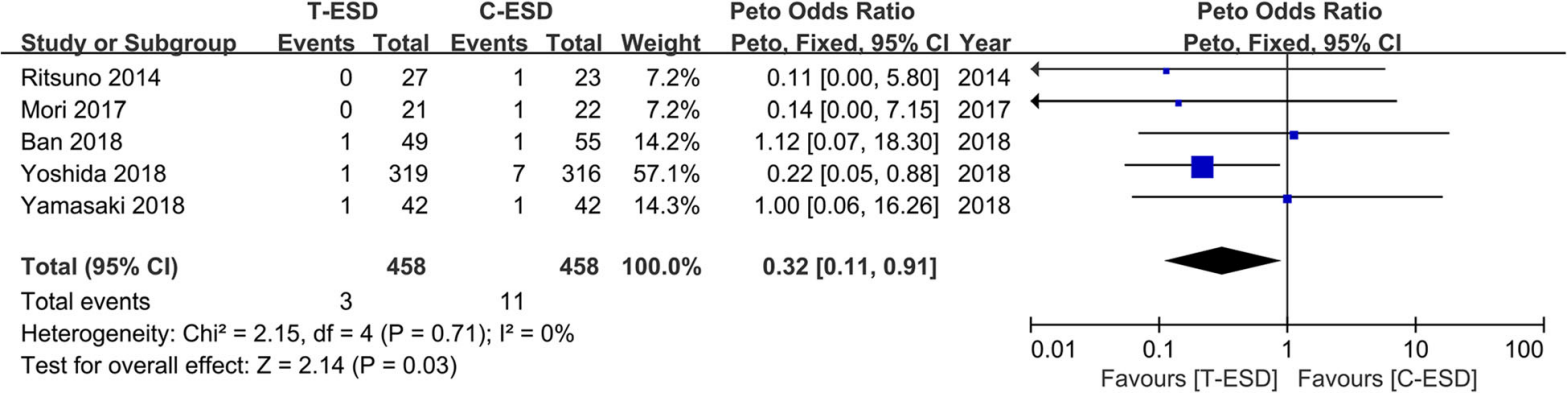
Forest plot of perforation

After the studies were removed, in which the incidence of both groups was 0, we finally pooled estimate 5 studies including 916 patients. Perforation was observed in 3 of 458 patients in the T-ESD group and in 11 of 458 patients in the C-ESD group. When the data were pooled, there was a significant difference in the incidence of perforation between the two groups. Compared to C-ESD, T-ESD was associated with lower incidence of perforation (OR = 0.32, 95% CI 0.11, 0.91, *I*^2^ = 0%, *P* = 0.03). There was no statistical heterogeneity (Fig. 5).

#### Delayed bleeding

**Fig. 6:**
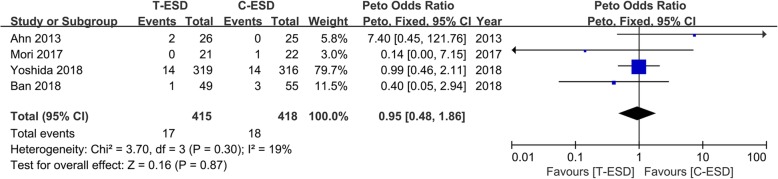
Forest plot of delayed bleeding

All studies reported the incidence of delayed bleeding including 1007 patients. After the studies were removed, in which the incidence of both groups was 0, we finally pooled estimate four studies including 833 patients. The pooled estimate on the fixed effect model indicated no significant difference in delayed bleeding between the two groups (OR = 0.95, 95% CI 0.48, 1.86, *I*^2^ = 19%, *P* = 0.87). There was slight statistical heterogeneity (Fig. 6).

### Subgroup analysis

**Fig. 7:**
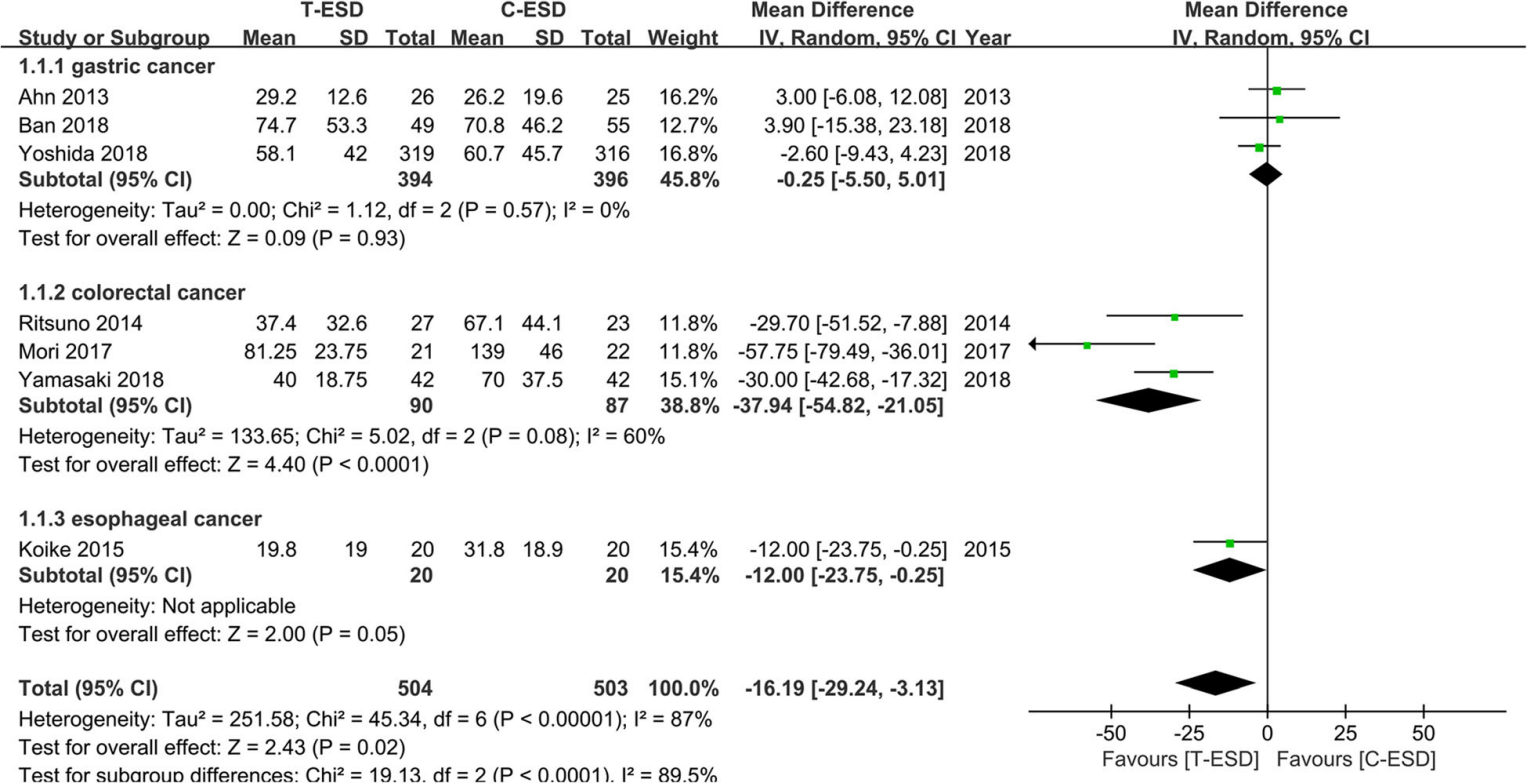
Forest plot of procedure time of subgroup analysis according to the location of lesion

Because studies evaluating patients with lesions in different locations were combined in the present meta-analysis, we performed a subgroup analysis according to the lesion location: gastric neoplasms, colorectal neoplasms, and esophageal neoplasms. The pooled estimate of procedure time demonstrated that there was no significant difference between the T-ESD and C-ESD groups in the gastric neoplasms group (MD = − 0.25, 95% CI − 5.5, 5.01, *I*^2^ = 0%, *P* = 0.93), while in colorectal neoplasms, the procedure time was significantly shorter in the T-ESD group than in the C-ESD group (MD = − 37.94, 95% CI − 54.82, − 21.05, *I*^2^ = 60%, *P* < 0.0001). Heterogeneity was still significant. Only one study in the esophageal neoplasms group suggested that there was no statistically significant difference between the T-ESD and C-ESD groups (Fig. 7).

We performed a sensitivity analysis and removed one study at a time, but the heterogeneity did not change significantly.

## Discussion

This systematic review and meta-analysis compare T-ESD to C-ESD. The pooled results demonstrate that T-ESD and C-ESD were similarly effective in en bloc resection, complete resection, and delayed bleeding. However, we provide evidence that T-ESD has significant advantages in that it is associated with a lower incidence of perforation. During the operation, traction technology can effectively reduce the operation time.

ESD has become the first-line treatment of superficial gastrointestinal neoplasms with high en bloc resection and curative resection rates. The safety of the ESD process is especially important in a variety of clinical situations. According to National Cancer Center Hospital of Japan [25], delayed bleeding occurred in 6% of patients; perforation is the complication of greatest concern, and it occurred in 3% of patients during the ESD procedure. In addition to the operator’s experience, a poor visual field of the cutting line can result in deeper tissue and submucosal vessels being vulnerable to injury and may be associated with a high risk of complication. Our study suggests that a traction system that facilitates direct visualization of the submucosal layer is effective for reducing the incidence of perforation. The study of Xie et al. [26] showed T-ESD to be beneficial for preventing muscular layer injury. This conclusion potentially confirms our result.

Previously, most studies in humans and animals have indicated that T-ESD is an appropriate alternative for C-ESD with shorter time [26–29]. Our study supports this finding. However, there is substantial heterogeneity in the outcome of procedure time. In the included studies, three articles concern colorectal neoplasms, three concern gastric neoplasms, and one concerns esophageal neoplasms, which may be a selection bias based on the anatomical characteristics. We know the difficulty level varies based on the location of lesions. Considering the differences in blood vessel size, fat levels, and angles, even in the same portion of the stomach, the degree of difficulty greatly differs [30]. For colorectal neoplasms, it is clear that the proximal colon is a more difficult location than the rectum and requires longer operation time. In the included studies of our meta-analysis, the difference of operator’s experience may be related to procedure time and result in a significant heterogeneity. Furthermore, differences in devices and equipment such as in the endoscope, knife, and traction method may be associated with heterogeneity to some degree.

Due to the complexity of ESD, the success of ESD procedure depends to some extent on abundant endoscopic experience and on the skill of endoscopist. Gotoda et al. [31] reported that at least 40 ESD procedures are needed for a trainee endoscopist to overcome the learning curve and gain proficiency in this technique. Operators who lack of ESD experience will lead to higher incidence of procedural complications such as bleeding and perforation. In addition, considering the anatomical characteristics of different organs, the complications are related to patient groups with the lesions located in different locations. The esophageal diameter is narrower than other organs, the stomach is a J-shaped organ that can appear various shapes with peristalsis and is divided into five areas: the cardia, the fundus, the body, the antrum, and the pylorus, the colorectum with thin intestinal wall and small angulated lumen. When the lesions are located in easy-to-operate locations such as the rectum and the lower part of the stomach, there will be fewer complications than in other locations. Therefore, it is important to evaluate the effect of ESD and traction methods according to the lesion location.

To obtain better visibility, a variety of traction methods have been applied to facilitate the ESD procedures, such as the clip with line method [12], the magnetic anchor method [32], the double-endoscope method [33], the internal traction method [14, 30], and the external grasping forceps method [13]. However, each method has its own advantage and disadvantage. The clip with line is simple and helpful in almost all gastrointestinal neoplasms, but the direction of traction is limited, and the endoclip is easily detached from the specimen. For magnetic anchor ESD, the direction and degree of traction can be easily controlled by changing the location of the external magnet. The disadvantages of this traction method are that the strength is attenuated with the amount of abdominal fat and the internal magnet requires additional coating to prevent damage to the human body. The double-scope technique can adjust the direction of traction by maneuvering the endoscope, changing the angle, and inserting or retracting the grasping forceps. However, this method still has shortcomings. First, two endoscopes will interfere with each other. Second, this method requires adequate space for the placement of two light sources and the occurrence of optical interference. The internal traction includes several methods, such as S-O clip, medical ring, clip modifications, rubber strips, and clip-band. This technique can apply to any direction, but it is difficult to control the traction direction and requires special devices and equipment. Through external grasping forceps method, the direction of traction can be easily adjusted by pushing and pulling the forceps, and no assistant is required to hold the forceps during the ESD procedure. However, the forceps are not flexible, and it is sometimes difficult to anchor the distal edge of the lesion. Hence, future randomized clinical trials comparing the different traction techniques are warranted to help define the suitable traction method for different locations.

Several limitations are presented in this study. First, we included seven articles in English, six of them from Japan and one from Korea, so generalizing the results to other races should be done with caution. Second, the number of included studies is limited, and most of them are small samples. The only study with a large sample size accounts for a large weight in Figs. 2, 3, 5, and 6, so the credibility of the result may be affected. Third, due to the features of the operative procedure, the risk of failure to apply blinding should be considered. Fourth, we could not separately evaluate a particular traction technique because the selected trials used different traction methods. Fifth, due to the high success rate of en bloc resection and complete resection in the ESD procedure, we cannot get a meaningful conclusions. Therefore, we expect that there will be more large sample studies to focus on en bloc resection and complete resection in the future. Finally, the analysis of the long-term oncological outcomes were not available due to the lack of insufficient information.

## Conclusion

This systematic review and meta-analysis demonstrate the traction-assisted endoscopic submucosal dissection is superior to conventional ESD for Asians with superficial gastrointestinal neoplasms. Traction ESD effectively reduces the perforation rate and shortens the operation time. Future multi-center (including European populations), randomized controlled trials of larger sample size and long-term outcomes of T-ESD are awaited to further firm the conclusion.

## Data Availability

All data generated or analyzed during this study are included in this published article.

## Appendix

Embase:

#1 ‘endoscopic submucosal dissection’/exp. OR ‘Submucosal Dissection*’:ti,ab OR ‘endoscopic Dissection*’:ti,ab OR ‘endoscopic mucosal dissection*’:ti,ab OR ‘Dissection*, Endoscopic Submucosal’:ti,ab OR ‘Endoscopic Submucosal Dissection*’:ti,ab OR ‘Submucosal Dissection*, Endoscopic’:ti,ab OR ESD:ti,ab

#2 Pull*:ti,ab OR Drag*:ti,ab OR Haul*:ti,ab OR tow*:ti,ab OR traction:ti,ab OR guid*:ti,ab

#3 Clip*:ti,ab OR Hemoclip*:ti,ab OR endoclip*:ti,ab OR thread*:ti,ab OR string*:ti,ab OR snare*:ti,ab OR magnetic:ti,ab OR Anchor*:ti,ab OR sinker*:ti,ab OR band*:ti,ab OR robot*:ti,ab OR EndoLifter*:ti,ab OR pulley*:ti,ab OR Spring*:ti,ab OR ‘dental floss*’:ti,ab OR (external NEAR/2 forcep*):ti,ab OR ‘internal traction’:ti,ab OR pre-looping OR prelooping OR ‘suture material’:ti,ab OR (double NEAR/2 scope):ti,ab OR ‘transnasal endoscope’:ti,ab OR (percutaneous NEAR/2 traction):ti,ab OR ‘endoscopic surgical platform’:ti,ab OR ‘steerable grasper*’:ti,ab OR ‘retraction strip*’:ti,ab OR ‘medical ring*’:ti,ab OR (robotic NEAR/2 manipulator):ti,ab OR ‘cross-counter technique’:ti,ab

#4 #2 OR #3

#5 #1 AND #4

PubMed

#1 “Submucosal Dissection”[Title/Abstract] OR “Submucosal Dissections”[Title/Abstract] OR “endoscopic Dissection”[Title/Abstract] OR “endoscopic Dissections”[Title/Abstract] OR “endoscopic mucosal dissection”[Title/Abstract] OR “endoscopic mucosal dissections”[Title/Abstract] OR “Dissection, Endoscopic Submucosal”[Title/Abstract] OR “Dissections, Endoscopic Submucosal”[Title/Abstract] OR “Endoscopic Submucosal Dissection”[Title/Abstract] OR “Endoscopic Submucosal Dissections”[Title/Abstract] OR “Submucosal Dissection, Endoscopic”[Title/Abstract] OR “Submucosal Dissections, Endoscopic”[Title/Abstract] OR ESD[Title/Abstract]

#2 Pull*[Title/Abstract] OR Drag*[Title/Abstract] OR Haul*[Title/Abstract] OR tow*[Title/Abstract] OR traction[Title/Abstract] OR guided[Title/Abstract] OR guidance[Title/Abstract] OR guide[Title/Abstract]

#3 Clip*[Title/Abstract] OR Hemoclip*[Title/Abstract] OR endoclip*[Title/Abstract] OR “dental floss*”[Title/Abstract] OR “external forcep*”[Title/Abstract] OR “external grasping forcep*”[Title/Abstract] OR “internal traction”[Title/Abstract] OR “Thread* traction”[Title/Abstract] OR string*[Title/Abstract] OR snare*[Title/Abstract] OR pre-looping[Title/Abstract] OR prelooping[Title/Abstract] OR “suture material”[Title/Abstract] OR “double scope”[Title/Abstract] OR “double channel scope”[Title/Abstract] OR “transnasal endoscope”[Title/Abstract] OR magnetic[Title/Abstract] OR Anchor*[Title/Abstract] OR “percutaneous traction”[Title/Abstract] OR “percutaneous transgastric traction”[Title/Abstract] OR sinker*[Title/Abstract] OR band*[Title/Abstract] OR “endoscopic surgical platform”[Title/Abstract] OR “ring thread*”[Title/Abstract] OR “ring shaped thread*”[Title/Abstract] OR “steerable grasper*”[Title/Abstract] OR “retraction strip*”[Title/Abstract] OR “medical ring*”[Title/Abstract] OR “robotic suture manipulator”[Title/Abstract] OR “robotic manipulator”[Title/Abstract] OR EndoLifter*[Title/Abstract] OR Spring*[Title/Abstract] OR “cross-counter technique”[Title/Abstract] OR pulley*[Title/Abstract] OR robot*[Title/Abstract]

#4 #2 OR #3

#5 #1 AND #4

Cochrane

#1 Submucosal Dissection*:ti,ab,kw or endoscopic Dissection*:ti,ab,kw or endoscopic mucosal dissection*:ti,ab,kw or Dissection*, Endoscopic Submucosal:ti,ab,kw or Endoscopic Submucosal Dissection*:ti,ab,kw or Submucosal Dissection*, Endoscopic:ti,ab,kw or ESD:ti,ab,kw

#2 Pull*:ti,ab,kw or Drag*:ti,ab,kw or Haul*:ti,ab,kw or tow*:ti,ab,kw or traction:ti,ab,kw or guid*:ti,ab,kw

#3 Clip*:ti,ab,kw or Hemoclip*:ti,ab,kw or endoclip*:ti,ab,kw or string*:ti,ab,kw or snare*:ti,ab,kw or pre-looping:ti,ab,kw or prelooping:ti,ab,kw or magnetic:ti,ab,kw or Anchor*:ti,ab,kw or sinker*:ti,ab,kw or band*:ti,ab,kw or EndoLifter*:ti,ab,kw or Spring*:ti,ab,kw or pulley*:ti,ab,kw or robot*:ti,ab,kw or “dental floss*”:ti,ab,kw or “external forcep*”:ti,ab,kw or “external grasping forcep*”:ti,ab,kw or “internal traction”:ti,ab,kw or “Thread* traction”:ti,ab,kw or “suture material”:ti,ab,kw or “double scope”:ti,ab,kw or “double channel scope”:ti,ab,kw or “transnasal endoscope”:ti,ab,kw or “percutaneous traction”:ti,ab,kw or “percutaneous transgastric traction”:ti,ab,kw or “endoscopic surgical platform”:ti,ab,kw or “ring thread*”:ti,ab,kw or “ring shaped thread*”:ti,ab,kw or “steerable grasper*”:ti,ab,kw or “retraction strip*”:ti,ab,kw or “medical ring*”:ti,ab,kw or “robotic suture manipulator”:ti,ab,kw or “robotic manipulator”:ti,ab,kw or “cross-counter technique”:ti,ab,kw

#4 #2 OR #3

#5 #1 AND #4

## Abbreviations

C-ESD: Conventional endoscopic submucosal dissection
EMR: Endoscopic mucosal resection
ESD: Endoscopic submucosal dissection
GI: Superficial gastrointestinal
RCTs: Randomized controlled trials
T-ESD: Traction-assisted endoscopic submucosal dissection
